# Improving Effects of Peripheral Nerve Decompression Microsurgery of Lower Limbs in Patients with Diabetic Peripheral Neuropathy

**DOI:** 10.3390/brainsci13040558

**Published:** 2023-03-26

**Authors:** Fukai Ma, Guangyu Wang, Yiwei Wu, Bingran Xie, Wenchuan Zhang

**Affiliations:** Department of Neurosurgery, Shanghai Ninth People’s Hospital, Shanghai Jiao Tong University School of Medicine, Shanghai 200011, China; fkma@fudan.edu.cn (F.M.);

**Keywords:** diabetic peripheral neuropathy, peripheral nerve decompression microsurgery, nerve conduit

## Abstract

Background: Peripheral nerve decompression microsurgery can relieve nerve entrapment and improve the symptoms of DPN. However, postoperative tissue adhesion will produce new pressure on the nerves, affecting the surgical efficacy. In this study, a nerve conduit was used in the peripheral nerve decompression microsurgery to prevent postoperative adhesions, and the role of the nerve conduit in surgical nerve decompression was explored. Methods: A total of 69 patients with DPN were recruited and randomly divided into three groups: the nerve conduit group, conventional surgery group, and control group. Two weeks before surgery and 6 months after surgery, patients in each group were clinically tested using the visual analog scale (VAS) score, neurophysiological test, Toronto clinical scoring system (TCSS) score, and two-point discrimination (2-PD) test. Results: The patients’ symptoms in the nerve conduit group were relieved to varying degrees, and the relief rate reached 90.9%; the treatment efficacy was higher than that in the other groups. The postoperative nerve conduction velocity (NCV) in the two surgical groups was significantly higher than that before the surgery, and the difference between the nerve conduit group and the conventional surgery group was statistically significant (*p* < 0.05). For the 2-PD test, there was a statistically significant difference between the two surgical groups (*p* < 0.05). The TCSS score in the two surgical groups was significantly higher than that in the control group (*p* < 0.01). There was a significant difference in the TCSS scores between the nerve conduit group and the conventional surgery group (*p* < 0.05). Conclusions: The nerve conduit could further improve the efficacy of peripheral nerve decompression microsurgery in the treatment of DPN.

## 1. Introduction

Diabetic peripheral neuropathy (DPN) is one of the most common chronic complications of diabetes, with an incidence rate of as high as 50–60% [1,2]. It is also the main cause of foot ulcers, infections, and amputations [3,4]. DPN has long been considered “progressive and irreversible”. It is mainly manifested as pain, numbness, and paresthesia in the glove-sock-like distribution area at the end of the extremities, seriously influencing the patients’ quality of life, and results in a heavy social burden [5,6,7]. In recent years, it has generally been demonstrated to be the result of a combination of factors including long-term hyperglycemia caused by metabolic disorders, microcirculation disorders, and neuroischemia, and hypoxia [8]. The pathogenesis of DPN is complex and remains unclear. It involves changes in osmotic pressure, glycation end products, neuromicroangiopathy, autoimmune responses, oxidative stress responses, and deficiencies of neurotrophic and nerve growth factors.

Traditional treatment of DPN mainly includes controlling blood sugar and analgesia, metabolic regulation, and improvement in the microcirculation, in order to eliminate ischemia and hypoxia as well as enhance nerve conduction function [9]. Other methods have also been reported such as the consumption of antioxidants, supplementation of nerve growth factors, and the administration of immunosuppressants [10,11]. However, there is currently no specific and satisfactory therapeutic method in clinical practice [12]. Professor Dellon found that the sensitivity of peripheral nerves to compression increased during diabetes, and demonstrated that the symptoms of DPN were caused by nerve compression, and after taking the lead in the treatment of DPN with peripheral nerve decompression microsurgery, the surgery was effective, and the course of DPN changed [13]. In recent years, according to Dellon et al.’s findings [14,15], several hospitals have applied peripheral nerve decompression microsurgery to treat DPN. Satisfactory efficacy of peripheral nerve decompression microsurgery assists patients with DPN to restore their feelings and relieve pain [16].

However, after surgery, nerves may adhere to the surrounding tissues, hindering the extension of the axons to the distal end. At the late stage, it may cause new compression on the nerve, affecting the recovery of nerve function, and the symptoms are not significantly improved, and new symptoms may even occur [17]. To prevent the adherence of nerves to the surrounding tissue or the scar tissue formation caused by recompression of nerves, in the present study, we used a nerve conduit to wrap the nerve after surgery. Six months after the surgery, the effects of the nerve conduit were evaluated by the Toronto clinical scoring system (TCSS) score, visual analog scale (VAS) pain score, neuronal electrophysiology, and two-point discrimination (2-PD) test.

## 2. Materials and Methods

### 2.1. General Data

A total of 69 patients who were diagnosed with type 2 diabetes and treated in the Ninth People’s Hospital Affiliated with Shanghai Jiaotong University School of Medicine (Shanghai, China) between March 2019 and March 2022 were enrolled. The study population included 35 males and 34 females, with the mean age of 63.8 ± 10.7 years old, the time of diagnosis of type 2 diabetes was 10.4 ± 4.7 years, and the time to onset of DPN symptoms was 15.9 ± 7.6 months. Patients presented with spontaneous neuralgia below the knees and on the bottom of the foot with sensory disturbance. Electromyography (EMG) confirmed peripheral neuropathy in the lower limbs. Neural Tinel’s sign was positive, and patients had no severe edema of the feet. Peripheral neuropathy in the lower limbs caused by cervical and lumbar spine lesions, infectious polyneuritis, chronic alcoholism, drug poisoning, thyroid disease, tumors, cerebrovascular sequelae, and peripheral vascular disease were excluded in all patients. Patients were randomly divided into three groups: the nerve conduit group, conventional surgery group, and control group including 23 cases in each group. Patients in each group had not received regular treatment for more than 3 months before the experiment. After the start of the experiment, all patients received regular diabetic treatment, hemoglobin A1c (HbA1c) ≤ 7.5%. In the nerve conduit group and conventional surgery group, the fasting blood glucose was controlled at about 8 mmol/L for at least 2 weeks before surgery. This study was conducted in accordance with the Declaration of Helsinki.

### 2.2. Treatment Method

The nerve conduit was obtained from Beijing TianXinFu Medical Appliance Co., Ltd. (Beijing, China). The diameter of the nerve conduit was 3 mm, with the length of 1.3 cm. Control group program: epalrestat, lipoic acid capsules, beraprost sodium tablets, and mecobal tablets. Conventional surgery group plan: the classic Dellon lower extremity triple nerve decompression surgery was performed, and three incisions were made in the lower extremity: the first surgical incision was located 1.5 cm below the fibular head, a 2.5 cm oblique incision was made, and the main trunk of the common peroneal nerve was exposed. The fascia and the peroneus longus tendon were incised to fully expose the superficial and deep branches of the common peroneal nerve, in which a part of the tendon was cut at the nerve entrapment site to release the common peroneal nerve, and the epineurium was released along the surface of the common peroneal nerve. Then, a longitudinal 2.5 cm incision was made on the first and second toes of the dorsum of the foot, the deep peroneal nerve was freed by incising the skin subcutaneously, and the deep peroneal epineurium was released throughout the distal end. Finally, an arc-shaped incision was made at the tarsal canal, about 6 cm in length, in which the skin and flexor retinaculum were incised, the posterior tibial artery, vein, and their branches were separated and protected, and the tibial nerve and its branches were separated below it. This was freed and fully decompressed along the direction of the nerve, and the epineurium was released along the nerve surface. For the nerve conduit group, after the nerve was decompressed, it was wrapped by the nerve conduit. Neurolysis and decompression surgeries were performed under a microscope. Data on the lower extremity with the most serious symptoms were collected. This was a prospective randomized two-blind trial. The following test was completed by the same researcher who was blinded to the treatment.

### 2.3. TCSS Score

TCSS scores were calculated for patients 2 weeks before surgery and 6 months after surgery. The TCSS score is basically composed of three parts: neurological symptoms, neural reflexes, and sensory function scores; neurological symptoms include lower extremity numbness, pain, needle-like sensation, fatigue, unsteady walking, in which 0 and 1 point is considered for the absence and presence of neurological symptoms, respectively, in total accounting for 6 points; nerve reflexes include ankle and knee reflexes, in which both sides are scored separately, involving 2 points for disappearance of reflexes, 1 point for weakened reflexes, and 0 point for normal reflexes, in total accounting for 8 points; sensory function includes pain, temperature, touch pressure, vibration, and position sense of the big toe, involving 1 point for abnormality and 0 point for normality, in total accounting for 5 points. The total score was 19 points.

### 2.4. VAS Score Measurement

The patients’ level of spontaneous pain was evaluated using the VAS score measurement. The VAS is one of the oldest and most reliable scales for measuring the severity of pain [18]. Two weeks before surgery and every 2 months after surgery, pain was assessed by the VAS score, the pain level gradually increased from 0 to 10 points, and the curative effect was assessed by the decrease in the VAS score after treatment.

### 2.5. Nerve Conduction Velocity (NCV)

An electromyograph (model DK-2740; Medtronic Co., Ltd., Copenhagen, Denmark) was used to detect NCV, in a quiet indoor environment, at a room temperature of 25 °C and skin temperature of 30 °C. Stimulation and recording were performed by the compliant surface electrodes. The NCVs of the common peroneal nerve and tibial nerve were recorded for the three groups 2 weeks before surgery and 6 months after surgery [19].

### 2.6. 2-PD Test

2-PD discrimination is the ability to discern that two nearby objects touching the skin are truly two distinct points, and not one. A baseline two-point discriminator (Fabrication Enterprises, New York, NY, USA) was used to perform the 2-PD test on patients 2 weeks before surgery and 6 months after treatment. During the neurological examination, patients were asked to close their eyes to avoid bias in the results. Two points of the skin of the hallux palm were stimulated with the separated feet of a two-point limen tool, applying pressure on the skin. If the patient could feel two points, the distance between the two feet could be shortened by adjusting the tool until the patient could feel one point, then, the distance was recorded. In this study, the great toe two-point discrimination of the hallux palm was higher than 9 mm [20].

### 2.7. Statistical Analysis

SPSS 13.0 software (IBM, Armonk, NY, USA) was used to perform statistical analysis. Data were presented as the mean ± standard deviation (x ± s) and analyzed using the paired sample *t*-test, covariance analysis, and repeated measures analysis of variance (ANOVA). *p* < 0.05 was considered as statistically significant.

## 3. Results

### 3.1. General Data

**Figure 1:**
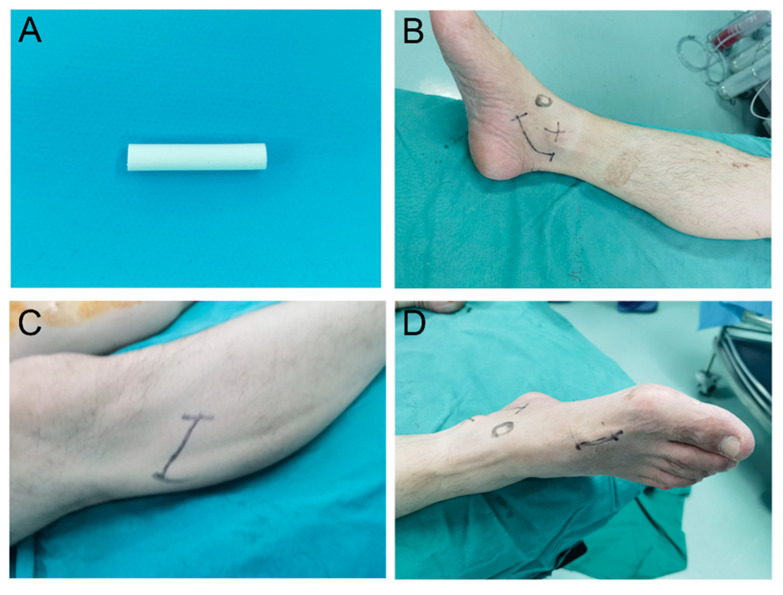
Illustration of the nerve conduit and incisions. (**A**) Photograph of the nerve conduit. (**B**) Photograph of the third surgical incision. (**C**) Photograph of the first surgical incision. (**D**) Photograph of the second surgical incision.

All 69 patients successfully completed the surgery (Figure 1), and in the review process, one patient in the nerve conduit group was excluded from the study due to the loss to follow-up. It was recorded as effective if the patient experienced a relief of symptoms (Table 1). The remission rate in the nerve conduit group, conventional surgery group, and control group was 90.9%, 82.6%, and 26.1%, respectively (Table 2). The surgical efficacy in the nerve conduit group was the highest among the three groups (Table 2). No peripheral nerve injury occurred after surgery, the incisions were healed well, and no ulcer or amputation was found. One patient in the nerve conduit group underwent surgery again at 6 months after the recurrence of symptoms. It was found that the nerve conduit was completely degraded when the patient underwent surgery again.

### 3.2. TCSS Score

**Figure 2:**
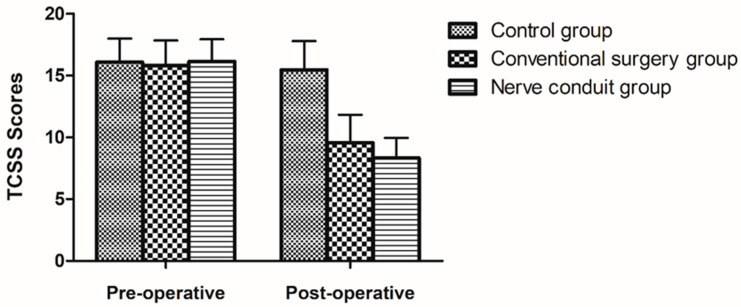
The figure displays the records of the TCSS scores in all groups (mean ± SD).

TCSS scores were calculated for the statuses of 2 weeks before surgery and 6 months after surgery. The average preoperative TCSS score in the nerve conduit group was 16.14 ± 1.81 points, 8.32 ± 1.64 points after treatment, and the difference was statistically significant (*p* < 0.05). The average preoperative TCSS score in the conventional surgery group was 15.83 ± 2.01 points, 9.57 ± 2.25 points after surgery, and the difference was statistically significant (*p* < 0.05). The average TCSS score in the control group was 16.09 ± 1.9 points before treatment, 15.48 ± 2.31 points after treatment, and the difference was not statistically significant (*p* > 0.05). The above-mentioned results suggest that the curative effect in the two surgical groups was better than that in the control group. There was a significant difference between the two surgical groups before and after treatment (*p* < 0.05), and it was revealed that the surgical efficacy in the two surgical groups was greater than that in the control group (Table 3) (Figure 2).

### 3.3. VAS Score

**Figure 3:**
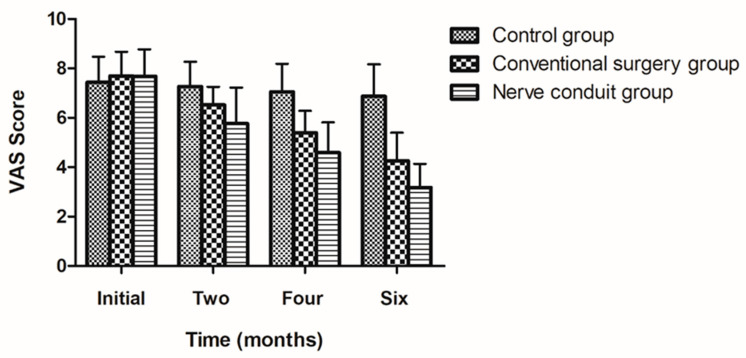
The figure illustrate the results of the VAS levels at different time points for each group (mean ± SD).

According to the degree of improvement in the patients’ symptoms, the VAS score was used to evaluate the pain level before and after treatment. There was no significant difference in the preoperative VAS score among the three groups. The VAS score in the nerve conduit group showed a gradually decreasing trend after surgery, while the VAS score in the control group did not significantly change during the follow-up period. The VAS score in the conventional surgery group was between that in the nerve conduit group and the control group. The VAS score in the two surgery groups was significantly lower than that in the control group at 6 months after surgery (*p* < 0.01). At 6 months after surgery, the VAS score in the nerve conduit group decreased from 7.68 ± 1.09 to 3.18 ± 0.96 points before and after surgery, and the difference was statistically significant (*p* < 0.05). The VAS score in the conventional surgery group decreased from 7.7 ± 0.97 to 4.26 ± 1.14 points before and after surgery. In comparison, there was no significant difference in the control group before and at 6 months after surgery for the VAS score (*p* > 0.05). There was a statistically significant difference in the VAS score between the nerve conduit group and the conventional surgery group (*p* < 0.05). The above-mentioned results suggest that nerve decompression surgery could reduce symptoms in patients, especially with the application of a nerve conduit (Table 4) (Figure 3).

### 3.4. Electrophysiological Evaluation

**Figure 4:**
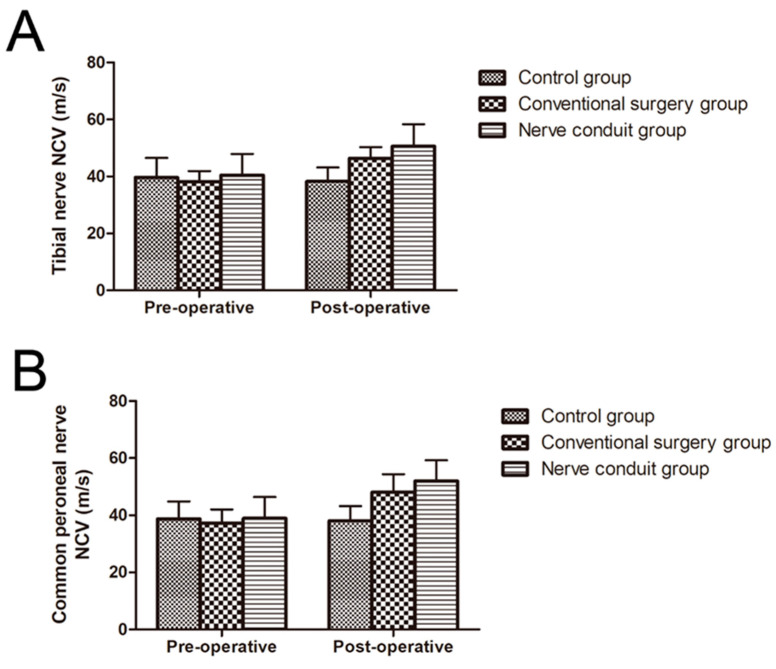
Pre-operative and post-operative NCVs of patients in all groups (mean ± SD). (**A**) Nerve conduction velocity of the tibial nerve. (**B**) Nerve conduction velocity of the common peroneal nerve.

The NCVs of 68 patients were recorded. As presented in Table 5, different degrees of improved NCV could be observed in the majority of patients with DPN in the nerve conduit group and conventional surgery group. The NCV in the nerve conduit group was 40.4 ± 7.5 m/s before surgery and 50.6 ± 7.8 m/s after surgery for the tibial nerve, and 38.9 ± 7.5 m/s before surgery and 52 ± 7.3 m/s after surgery for the common peroneal nerve, which was the highest velocity among all groups, indicating that the NCV of patients after surgery was significantly higher than that before surgery (*p* < 0.05). At 6 months after nerve decompression surgery, the NCV in the conventional surgery group increased from 38.1 to 46.3 m/s for the tibial nerve, and 37.2 to 48 m/s for the common peroneal nerve, which was higher than that in the control group and lower than that in the nerve conduit group, and the difference was statistically significant (*p* < 0.05) (Figure 4).

### 3.5. 2-PD Test

**Figure 5:**
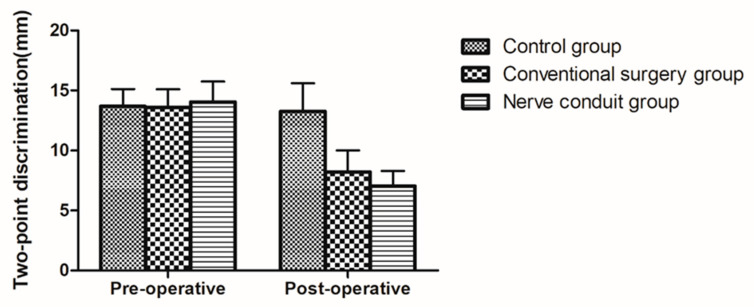
Diagram of 2-PD tests showing the changes in the three groups (mean ± SD).

In the two surgical groups, the 2-PD values significantly decreased half a year after surgery compared with those before surgery (*p* < 0.05). In the control group, however, no significant change in 2-PD values was noted after half a year (*p* > 0.05). Compared with the control group, the difference in 2-PD values before and after surgery in the two surgical groups was statistically significant (*p* < 0.05). Compared with the conventional surgery group, the difference in 2-PD values before and after treatment in the nerve conduit group showed a better improvement, and the difference was statistically significant (*p* < 0.05) (Table 6 and Figure 5).

## 4. Discussion

It is noteworthy that DPN is a frequent complication of diabetes and a major cause of morbidity and increased mortality, and has been shown to be associated with significant reductions in the overall quality of life, increased levels of anxiety and depression, sleep impairment, and greater gait variability [21,22,23]. In the absence of effective treatment, patients with diabetes may develop to progressive and irreversible loss of foot sensation, which may further lead to loss of balance in walking, easy falling and injuries, and even fractures. At the same time, DPN patients are prone to various painless injuries including scalds, foot ulcers, infections, gangrene, and even amputation, seriously influencing the patients’ quality of life [24,25,26].

Under normal circumstances, blood sugar enters the nerves to provide energy and is converted into fructose. A high blood sugar level causes fructose accumulation in the peripheral nerves of diabetic patients. The molecular formula of fructose determines its tendency to bind water. Therefore, water is sucked into the nerve, causing the nerve to swell, expanding its volume, stimulating the axons to swell, and is easy to compress [24,27]. An excessive amount of fructose impedes axoplasmic transport, thereby hindering the transport of lipoproteins that are essential for nerve maintenance and repair, leading to demyelinate lesions, which are also chronic pathological consequences of elevated intraneural pressure [28,29]. The formation of terminal glycosylation products in the peripheral nerves and non-enzymatic glucose, combined with the intraneural collagen, may reduce nerve elasticity, increase tension, and decrease the sliding properties [30]. Thus, anatomical and physiological properties of the nerve make itself more susceptible to compression. There are multiple anatomical stenoses on the pathways of peripheral nerves supplying the fingers and toes from the spinal cord such as the common peroneal nerve through the peroneal canal and the tibial nerve through the tarsal canal [31]. For diabetic patients, when the swollen nerve is compressed through the anatomical stenosis of the limb, long-term chronic entrapment leads to epineurial microvascular ischemia and aggravated demyelination of peripheral nerves, forming a vicious circle and corresponding clinical symptoms [32].

In the present study, peripheral nerve decompression microsurgery was applied to relieve nerve compression at the site of anatomical stenosis [33]. Before surgery, there was no significant difference in gender, age, time of diagnosis, and fasting blood glucose level among the three groups (Table 1) (*p* > 0.05). In addition, patients in the nerve conduit group exhibited good postoperative results after the repair of peripheral nerves observed using a minimally invasive microscope, and their feelings were satisfactory. The TCSS score is a method for the diagnosis and prognosis of DPN through patient complaints and physical examination, and its high efficiency was previously confirmed [34]. The TCSS score can evaluate the severity of DPN and can also be used as an index for the evaluation of postoperative treatment efficacy. The results of the present study showed that the TCSS score was higher in the nerve conduit group than that in the other two groups, and the difference was statistically significant. Electrodiagnostic testing was not only used to identify patients eligible for surgery, but was also utilized as an objective marker to follow the progress of large nerve fiber function [19,35]. The present study revealed that NCVs were consistent with the TCSS scores. In this study, a higher rate of surgical efficacy was found in the nerve conduit group compared with that in the other two groups (Table 2).

Peripheral nerve decompression microsurgery can relieve nerve entrapment, restore axonal transport and blood supply in the nerve, relieve numbness and pain, and promote tissue repair, while it cannot eliminate peripheral neuropathy caused by abnormal glucose metabolism. Therefore, insulin should still be used to control blood sugar stability and to improve metabolic disorders in diabetic patients postoperatively.

In the present study, the diabetic peripheral nerve decompression microsurgery was utilized as a minimally invasive fibrous neurosurgery with short operation time and less pain for the patient [36]. Diabetic patients with typical symptoms of peripheral neuropathy such as pain, numbness, paresthesia, and the peripheral neuropathy caused by other factors were excluded. When the diagnosis of DPN is confirmed by neurophysiological examination, nerve decompression can be performed [37]. During surgery, it was found that the nerves were significantly compressed when passing through the above-mentioned channels. In addition, it was revealed that the nerves became pale yellow in the surgery, and their texture was softer than that of the normal nerves. It was considered that the nerves were compressed for a long time, and degenerated under the state of hyperglycemia. After incision of the epineurium, there was pale yellow fluid. The intraneural effusion was presumed due to compressed ischemia–hypoxic neurodegeneration and nerve edema, leading to local exudation, which in turn increased the intraneural pressure.

The ligament or fibrous tissue was cut to release the compressed part on the nerve pathway, the compression on the nerve was removed, the blood supply of the nerve was restored, and the nerve was allowed to slide with the movement of the adjacent joints, so the symptoms of numbness and pain were relieved [38]. With the prolongation of the time course of lesion development, necrosis of the innervated muscles may gradually occur, which may finally be fibrous tissues, and the nerve damage is irreversible [39]. Therefore, we recommend patients undergo surgery when they experience tingling and numbness in the feet. If a patient waits until there is muscle degeneration and necrosis, it is difficult to recover with surgery. In the present study, the follow-up results showed that peripheral nerve decompression in patients with DPN could alter the natural history of DPN, resulting in sensory recovery as well as a relief in symptoms such as burning, pain, and numbness.

In this study, we used a nerve conduit to protect the nerves in the surgical area during surgery, which could prevent the recurrence of nerve compression due to tissue adhesion after surgery. The nerve conduit manly consists of collagen extracted from bovine Achilles tendon tissue, and it is one of the most appropriate peripheral nerve repair materials. A collagen material is mainly recognized as a natural polymer material with excellent biocompatibility and biosafety [40,41,42]. When collagen scaffolds are used for the repair of peripheral nerve defects, they can maintain a scaffolding effect for a long-time, which is conducive to the growth and proliferation of nerve cells and gradually form new tissues to achieve the purpose of tissue repair and regeneration [43,44]. At the same time, the implanted material is an exogenous substance to the body, and if the implantation time is very long, it can easily cause a foreign body reaction in the tissue. Therefore, the degradation time is one of the major factors determining whether the product can meet the requirements of scaffolds for tissue engineering [45]. In one patient who underwent surgery 6 months later, we found that the nerve sheath was completely absorbed and degraded. The collagen scaffold used in this study has degradable properties, making it appropriate for tissue regeneration. In addition, it has a special pore structure that meets the requirements for cell growth and nutrient transport, thereby facilitating organizational reconstruction. However, there were some limitations in the study. In future works, we will enroll a greater number of patients and evaluate the long-term prognosis of the patients. Furthermore, serial sensory conduction will be tested in future works, which may provide more objectivity and credibility to the conclusions.

## 5. Conclusions

In summary, the results of this study confirmed that peripheral nerve decompression microsurgery in the lower extremity was effective for patients with DPN. Based on the current data, intraoperative application of the nerve conduit can further improve the efficacy of peripheral nerve decompression microsurgery in the treatment of DPN. This method may be a new treatment option for patients with DPN who cannot achieve adequate symptom relief via medications.

## Figures and Tables

**Table 1:** Demographic and clinical features of patients in different groups.

	Nerve Conduit Group	Conventional Surgery Group	Control Group
Mean age (years old)	66.8 ± 9.9	62.4 ± 10.4	62.2 ± 11.5
Female, n	10	12	12
Male, n	13	11	11
Duration of diabetes (years)	9.74 ± 4.3	11.35 ± 4.7	10.2 ± 5.0
Duration of pain (months)	16.3 ± 8.6	18.2 ± 7.7	13.1 ± 5.4

**Table 2:** Comparison of the curative effect among the three groups [n (%)].

	N	Efficient	Invalid
Nerve conduit group	22	20 (90.9)	2 (9.1)
Conventional surgery group	23	19 (82.6)	4 (17.4)
Control group	23	6 (26.1)	17 (73.9)

**Table 3:** Pre- and post-operative TCSS scores in different groups.

	Nerve Conduit Group	Conventional Surgery Group	Control Group	P (1, 2)
Pre-operative	16.14 ± 1.81	15.83 ± 2.01	16.09 ± 1.9	P1 < 0.05
Post-operative	8.32 ± 1.64	9.57 ± 2.25	15.48 ± 2.31	P2 < 0.01

P1: Nerve conduit group vs. conventional surgery group; P2: Conventional surgery group vs. control group.

**Table 4:** Comparison of VAS scores at different time points among the three groups.

	Baseline	Postoperative			P (1, 2)
		2 Months	4 Months	6 Months	
Nerve conduit group	7.68 ± 1.09	5.77 ± 1.45	4.59 ± 1.22	3.18 ± 0.96	P1 < 0.05
Conventional surgery group	7.7 ± 0.97	6.52 ± 0.73	5.39 ± 0.89	4.26 ± 1.14	P2 < 0.01
Control group	7.43 ± 1.04	7.26 ± 1.01	7.04 ± 1.15	6.87 ± 1.29	

P1: Nerve conduit group vs. conventional surgery group; P2: Conventional surgery group vs. control group.

**Table 5:** Comparison of NCV before and after surgery among the three groups.

	Nerve Conduit Group		Conventional Surgery Group		Control Group		P
	Pre-Operative	Post-Operative	Pre-Operative	Post-Operative	Pre-Operative	Post-Operative	
Posterior tibial nerve	40.4 ± 7.47	50.56 ± 7.78	38.1 ± 3.81	46.35 ± 3.93	39.64 ± 6.88	38.24 ± 4.93	P1 < 0.05, P2 < 0.01
Common peroneal nerve	38.91 ± 7.48	51.96 ± 7.28	37.18 ± 4.87	48.03 ± 6.33	38.7 ± 6.15	38.01 ± 5.17	P1 < 0.05, P2 < 0.01

P1: Nerve conduit group vs. conventional surgery group; P2: Conventional surgery group vs. control group.

**Table 6:** Results of the 2-PD test in the three groups.

	Nerve Conduit Group	Conventional Surgery Group	Control Group	P (1, 2)
Pre-operative	14.05 ± 1.7	13.61 ± 1.5	13.7 ± 1.43	P1 < 0.05
Post-operative	7.05 ± 1.25	8.22 ± 1.78	13.26 ± 2.34	P2 < 0.01

P1: Nerve conduit group vs. conventional surgery group; P2: Conventional surgery group vs. control group.

## Data Availability

The data that support the findings of this study are available upon request to the corresponding author.
